# The Human Face

**Published:** 1885-02

**Authors:** D. Y. Cliff


### The Human Face.

BY D. Y. CLIFF.

When man first detected that the voice, sight, hearing, smell, and taste were all situated in and emanated from the head, he looked upon it, and its contours and proportions became to him comparable and beautiful; he said, grandly: "It is the image of God!" How much does the rest of the body owe to carnal passions and "pride of might"? Admiration and appreciation have surely played a large part in our development. The intellect animal looks to the face--has it an idea of beauty? Do we recognize "beauty" in the brute creation from long inbred association, or have they themselves had a hand (or an eye) in it? The fact that it contains scarcely anything to cringe or terrify us, at first sight, would seem to prove this inbred familiarity. We find nature to be born *in* and *of* ourselves. There are more dangers in the artificial productions of man than in the structures of Nature. The eye reaches further than the weapon; and it is easier to fall from a window than from a tree.

Some say the national face does not change, its apparent differences being the result of fashion--costume, hat, hair, etc. For my part it seems that the history of each age is painted on the faces of its people. Parents would seem generally to anticipate

(or form) in fancy the realities of their offspring--probably unknowingly. I have on several occasions been struck by odd faces here and there which belonged to a past age. Some will, of course, smile at this. Once, *e. g.*, at a sham parliament in a Cheshire town, I saw an exact reproduction of the face (as generally represented) of the Georgian epoch of English history. The high cheeks, the ruddy skin, particularly the wide, low forehead, with its distinctive depression (almost) in the middle of the forehead where the head curves downward, the broad face, the peculiar "look," etc.

The face of Charles I. suggests his artistic taste, his theological thoughtfulness (so general then), and a proud indifference to vulgar rowdyism. He was to his age what "Farmer George" was to his, and the Prince of Wales is to his--types thereof--the men thereof bearing one of its varied educations, but the same generally under each disguise.

It would be a long subject to discuss the features of the different ages in English history and speculate upon them, and, perhaps, foreign to this journal. It is this feeling we have, this recognition of a fact, that hurts our fancies to see an ugly artist, a handsome slave, and sometimes to wonder at the beautiful eyes some of our domestic animals possess. We find an innate pleasure in gazing on a handsome face.

The above causes, no doubt, have lent a diversity to the face of woman which reacts on the man. The favorite type is "married up" in excess of others, and effectually impressed on the race. To this we may trace, probably, the widely diverged races of men, the Mongol, the Negro, the European, etc. The transmission of the family likeness, paternally and maternally, is interesting to reflect on. That was a scandalous remark, to me, I read, I think in your journal, about the passing admirations of a mother being stamped on her children's faces. Why is not the husband, the favorite brother, the sister, the mother, the father, etc., oftener reproduced, if that be the case--with the double chance ? It is remarkable, though, that the eldest child seems very often to retain the strongest family likeness. But the strong likeness of brothers and sisters is an argument against it. Perhaps this is largely owing to their catching each other's expressions of countenance; and this again explaining why the "younger end" often differ so decidedly from their elders—lack of asssociation. This same thing applies to nations; hence the force of the child's remark: "All Frenchmen seem to grin alike." A national contortion.

One would like to have seen the face of the Persians who made it part of their education to "speak the truth." We could have seen it! *Was* the Spartan stern in aspect who lived for his country's good? Was Deborah a Jewess in her look? Can we not read Byron's poetry in his face, and the heaviness of pondering judgments in Hallam's? Do you doubt, as you look at Nero's face, that he could fiddle while Rome burnt? And so on; a man's mind shines out of his countenance, the face in repose, or unanimated, is the generality of that individual's mind. And so we turn to look on the faces around us to-day. Are not the majority mere livers—mere nonentities? These will not remain in history, but they will form the nation's destiny!

Our souls were filled with sadness when we found inanity behind a lovely face. Nature lied to us! Do the choice minority conquer in the long run? It is one long fight.—*Journal of Science.*

